# Urinary metabolic modulation in human participants residing in Siachen: a 1H NMR metabolomics approach

**DOI:** 10.1038/s41598-022-13031-5

**Published:** 2022-05-31

**Authors:** Sonia Gandhi, Vijayakumar Chinnadurai, Kuntal Bhadra, Isha Gupta, Ratnesh Singh Kanwar

**Affiliations:** 1 Metabolomics Research Facility, Institute of Nuclear Medicine and Allied Sciences (INMAS), Lucknow Road, Timarpur, Delhi, 110054 India; 2 Cognitive Control and Machine Learning Centre, Institute of Nuclear Medicine and Allied Sciences, Delhi, 110054 India; 3 Department of Endocrinology and Thyroid Research Centre, Institute of Nuclear Medicine and Allied Sciences, Delhi, 110054 India

**Keywords:** Biochemistry, Biological techniques, Biomarkers

## Abstract

The main physiological challenge in high altitude environment is hypoxia which affects the aerobic metabolism reducing the energy supply. These changes may further progress toward extreme environment-related diseases. These are further reflected in changes in small molecular weight metabolites and metabolic pathways. In the present study, metabolic changes due to chronic environmental hypoxia were assessed using 1H NMR metabolomics by analysing the urinary metabolic profile of 70 people at sea level and 40 people at Siachen camp (3700 m) for 1 year. Multivariate statistical analysis was carried out, and PLSDA detected 15 metabolites based on VIP score > 1. ROC analysis detected cis-aconitate, Nicotinamide Mononucleotide, Tyrosine, Choline and Creatinine metabolites with a high range of sensitivity and specificity. Pathway analysis revealed 16 pathways impact > 0.05, and phenylalanine tyrosine and tryptophan biosynthesis was the most prominent altered pathway indicating metabolic remodelling to meet the energy requirements. TCA cycle, Glycine serine and Threonine metabolism, Glutathione metabolism and Cysteine alterations were other metabolic pathways affected during long-term high-altitude hypoxia exposure. Present findings will help unlock a new dimension for the potential application of NMR metabolomics to address extreme environment-related health problems, early detection and developing strategies to combat high altitude hypoxia.

## Introduction

The high-altitude environment's main physiological challenge is low oxygen and low-pressure levels resulting in hypoxia^1,2^. This causes reduced arterial partial pressure and oxygen saturation resulting in physiological hypoxic conditions to tissues and organs, leading to high-altitude pulmonary edema (HAPE) and High-altitude cerebral edema (HACE)^3–6^. This condition further reduces the energy supply and weakens aerobic metabolism^2^. Various metabolic pathway changes are involved during oxygen deficiency, which is reflected in low molecular metabolites changes^7,8^. In the case of environmental hypoxia, the major concern is to reduce the resulting damage to the biological systems^9^ as it has been reported that lack of reactive oxygen species and oxidative stress during hypoxia results in alterations in metabolic pathways^10,11^. Studies are being carried out to understand the pathophysiology of health-related issues due to hypoxia. However, its early diagnosis remains a challenge leading to delays in treatment and worsening the disease^12,13^. Early predictive biomarkers can help identify military personnel and people sensitive to environmental hypoxia, thereby helping initial screening and deciding the line of treatment^14,15^. Various studies have been conducted to understand the metabolic alterations under different hypoxic conditions. Gareth et al. reported activation of pathways associated with lipid, protein, and purine nucleotide metabolism in response to acute hypoxic exercise and recovery^16^. Mikrogeorgiou et al. carried out a metabolomics study of hypoxia–ischemia in mouse brain using hyperpolarized 13C to get real-time information on brain metabolism under hypoxic ischemia^17^. Liao et al. reported changes in metabolic pathways related to the inflammatory response, fatty acid transportation, bile acid metabolism, and heme metabolism changed under high altitude exposure^14^. O’Brien et al. examined the metabolomic response towards progressive exposure to environmental hypoxia in plasma of healthy individuals before and during an ascent to Everest Base Camp. Their findings indicated an increase in the rate of glycolysis and fat-store mobilization, decreased isoleucine and glucose, and increased lactic acid and circulating levels of free fatty acids (palmitic acid, linoleic acid, and oleic acid)^18^. Zhu et al. found that plasma levels of hypoxanthine, cysteinyl glycine, d-arabinol, l-threonine, 2-ketobutyric acid, and succinic semialdehyde increased under acute high-altitude hypoxia^19^.

Metabolomics is the technique that focuses on changes in the level of the metabolites under any kind of stress or diseased state and has been widely used to identify early predictive markers for hypoxia, pharmacology, medical sciences, and various other fields^20–29^. It is a top-down approach that provides a systemic understanding of an individual or population's biochemical and physiological status. On a global scale, metabolic profiling can identify metabolic changes in response to genetic differences, environmental influences and disease or drug perturbations. NMR spectroscopy has been extensively used to detect a wide range of low molecular weight endogenous metabolites in body fluids and tissue extracts. It is rapid, non-destructive, rich in quantitative information and allows simultaneous investigation of the predefined set metabolites^30,31^.

Currently, there is not much scientific knowledge that evaluates the metabolic modulation caused by long-term high-altitude acclimatization. The present study aims to identify early predictive urinary metabolic biomarkers for adaptations/maladaptation in human participants on long-term exposure to high altitudes and further understand the pathophysiological mechanisms involved in acclimatization to high altitude hypoxia^32,33^. The study further explores to bring more insight into the modulation in metabolic pathways that play a crucial role in high altitude acclimatization. For this purpose, the study employs the 1H-NMR spectroscopy-based metabolomics with multivariate and pathway analysis. The study fundamentally aims to open a metabolomics knowledge window for future studies so that new drug development and personalized medicine will evolve for extreme environmental hypoxia.

## Materials and methods

### Participants

**Figure 1:**
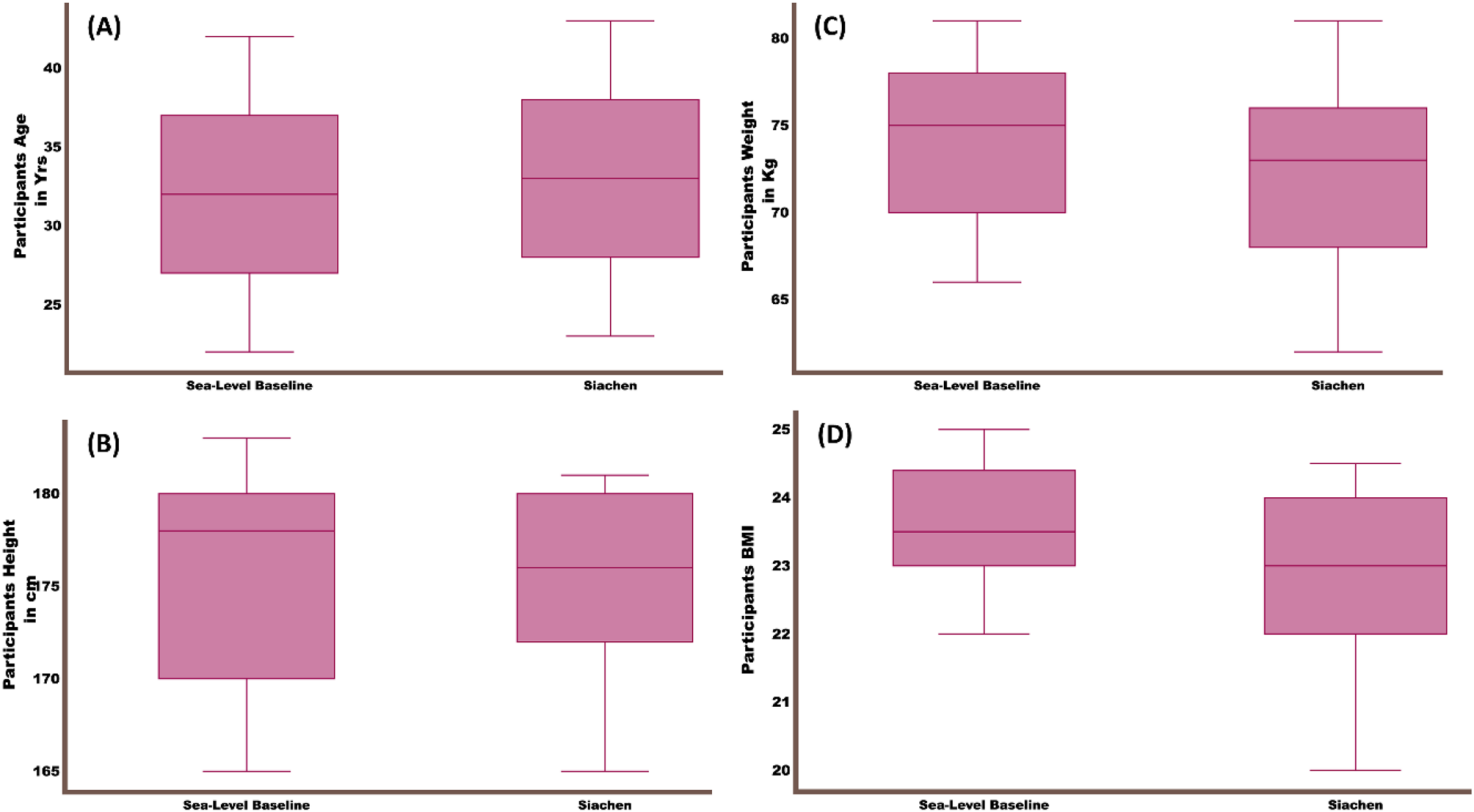
The summary of demographic and clinical data of the subjects.

The study protocol was approved by the institutional human ethics committee (IEC Ref No. IEC/DIPAS/C-11A/2), and all methods were performed following the relevant guidelines and regulations. Written informed consent was obtained from all the participants prior to the study. Seventy participants were recruited at sea level and the Siachen (altitude of 3700 m) for the study. Figure 1 plots the participants' age, weight, height, and BMI information. The study focuses on understanding metabolic mechanisms involved in acclimatization to high altitude hypoxia. Hence, all seventy participants were independently allowed to settle at sea level and Siachen for a year. Their urine samples were collected after 12 h fasting and stored at − 80 °C for further NMR acquisition and analysis. However, twenty-eight among seventy individuals who settled in Siachen had to move from Siachen altitude to other places due to extreme medical, logistic, and climate emergencies prevailing at that time. Hence, they could not wholly participate in the study, and their data were not included in the study analysis. Further, two Siachen group volunteers' NMR metabolites data were filtered out of the analysis as they had many outliers. More details about the outlier detection are explained in the later section.

### 1H-NMR spectroscopic analysis

Human urine samples were thawed, and 400 mL were mixed with 200 mL of deuterated 0.2 M sodium phosphate buffer solution (pH 7.4 containing one mM TSP prepared in D2O). The TSP acts as a chemical shift reference (d = 0) and D2O as a locking agent. The samples were then centrifuged at 5000 rpm for 5 min to remove particulate contaminants. The supernatant was poured into an NMR tube, and NMR spectral data were acquired on a Bruker Avance III HD spectrometer (Bruker, Germany) with a TXI probe operating at a frequency of 600.1 MHz at 298 K. A standard one-dimensional water pre-saturation pulse sequence (NOESYPR1D) was used to suppress water signal. A total of 64 scans (acquisition time 1.7 s/scan) were collected into 64 k data points and the spectral width of 9615.4 Hz with a relaxation delay of 2 s. An exponential function weighted the raw FIDs with a 0.3 Hz line broadening factor, followed by Fourier transformation. The resulting NMR spectra of each sample were manually corrected for phase and baseline using Bruker Topspin 3.6.4 (Bruker, Germany). The reference peak (TSP) was set to d = 0, followed by a visual inspection of the spectra from the control and high-altitude Siachen group. The data was pre-processed using topspin software, and individual metabolites were identified with the help of published literature using the Human Metabolome Database (HMDB).

### Data analysis

The modulation of each metabolite during high altitude hypoxia acclimatization is assessed by submitting both baseline controls and the Siachen group's NMR spectroscopy information to statistical analysis, which includes multivariate statistical analysis such as Principal Component Analysis (PCA), Partial least squares—discriminant analysis (PLS-DA), Sparse Partial least squares—discriminant analysis (sPLS-DA), Orthogonal projection to latent structures-discriminant analysis (OPLS-DA) and Random forest analysis.

### NMR spectral preprocessing and metabolites concentration estimation

At first, each participant's NMR spectrum was manually corrected for baseline and phase using TOPSPIN 3.6.4 (Bruker, Germany) as standard protocols our group followed and mentioned in earlier work by Koundal et al.^33^ and Tyagi et al.^34^. The corrected NMR spectra with the spectral range of δ 0.5–9.5 were binned in AMIX (Bruker, Biospin, Germany), and all the spectra were segmented into the binned region of equal width of 0.001 ppm. The water (4.5–5.0 ppm) and urea (5–6.0 ppm) NMR spectral information was eliminated from the binned NMR spectral information.

The performance of multivariate classification techniques entirely depends upon the quantitative analysis of the metabolites information from NMR spectra. Hence, this study employed a well-established quantitative metabolomics approach^35,36^, where thirty-six metabolites of interest are identified. Their concentrations were estimated by comparing preprocessed individual NMR spectrum with the pure components' standard spectral reference library information. The diagnostic peaks used to determine each metabolite's information were taken from the Human Metabolome Database—HMDB^37^ and Madison Metabolomics Consortium Database—MMCD^38^. The peaks of the selected metabolites were processed with spectral deconvolution with pre-fit reference spectra to match the reference spectrum to handle the overlapping spectral issues. Finally, the area of each metabolite spectra is normalised with the internal reference TSP standard's spectral area.

Before estimating metabolites, the NMR spectral data were preprocessed for zero filling and line broadening, chemical-shift referencing, phasing and baseline correction, line-shape correction, pH determination, and notch/water filtering. Further, to minimise the contribution from any outside noise influence on the estimated concentration information, every NMR spectral data processing and metabolite concentration estimation was cross-checked by an experienced NMR specialist. Then, every participant's metabolites data were checked for outliers. Mainly, the participant's metabolites data are considered an outlier and ignored if 30 out of 36 metabolites concentration value is more than three scaled median absolute deviations (MAD) away from the median of those metabolites total population^39^. This MAD based outlier removal has been implemented using preprocessing tools provided in Matlab 2021a (Mathworks). Two participants' metabolites data from Siachen groups were observed as outliers and removed from the subsequent data analysis. The baseline group did not have any outliers. Hence, the final sample sizes of the baseline and Siachen groups were 70 and 40, respectively. Finally, the estimated metabolites concentrations of each group were normalized to sum to reduce unwanted sample-to-sample variation. Each metabolic concentration is divided by the total sum of all other metabolic concentrations. The normalized metabolites concentrations were subsequently scaled using the auto-scaling approach. The auto-scaling, a similar method to Pareto scaling, takes standard deviation as the scaling factor in the denominator against Pareto scaling's process of taking the square root of the standard deviation as the scaling factor. Auto-scaling is ideally suited for scaling metabolites concentration as it performs unit variance scaling using the standard deviation as the scaling factor. All metabolites will have a single standard deviation, and thus the data are analyzed based on correlations instead of covariances, as is the case with centering. Furthermore, Pareto scaling is very sensitive to more enormous fold changes, and auto-scaling is not^40^.

### Univariate and multivariate data analysis

The preprocessed, normalized metabolites concentration information was subjected to the Student's t-test to identify differentially regulated metabolites between baseline and Siachen groups. The p and FDR values for significantly different metabolites were kept at 0.05. Then, the dataset of size 110 × 36 is subjected to various multivariate analyses that include PCA, PLS-DA, sPLS-DA, OPLS-DA and the random forest technique in Metaboanalyst 5.0^41^. At first, PCA was carried on for the preliminary assessment of systematic variation, similarities, and intrinsic clusters between the metabolic information of baseline and Siachen groups. Further, the weighted sum of PLS regression coefficients was calculated to identify important buckets with a maximum contribution to the separation of clusters in PLS-DA and assigned with the corresponding metabolite. The sPLS-DA and OPLS-DA were subsequently performed to improve the clustering of the datasets. For Sparse PLS-DA, the sparsity is introduced on the loading vectors through the lasso penalization.

Further, the Random forest techniques have been employed in this study to improve data analysis. This technique combines many decision trees constructed through the classification of each tree and voting the popular class with bootstrap sampling. The majority of the vote in the ensemble predicts the class in this approach. One-third of the samples are left out of bootstrapping during tree construction. A small cluster of input information is randomly taken as the node to construct a simple random forest with random features. The classification and regression tree (CART) approach grow each tree. In this study, the number of trees chosen was 5000.

### Validation

The present study assessed the predictive performance of multivariate models using cross-validation, R2/Q2/prediction accuracy and Receiver operating characteristic (ROC) curves with Area under curve (AUC) values. For the interpretation purpose, the closer R2, Q2 and accuracy values, the better the models' performance. In particular, the Q2 is better for model selection as it is less prone to model overfitting. Usually, the values of R2 and Q2 more than 0.5 denote the model is suitable. The VIP plots with a confidence level of 0.95 were used to select the variables. Finally, the predictive accuracy of both PLS-DA and random forest approaches has been evaluated with the different combinations of variables ranging from 2 to 36 selected from the VIP assessment. The ROC estimates the multivariate model's tradeoff between sensitivity (TP/(TP + FP)) and specificity (TN/(TN + FP), where TP, TN and FP are True positive, True Negative and False Positive. The points in ROC curves represent the predictive ability of the models, and Area under the ROC curve (AUC) brings a statistical summary of the model's performance.

### Metabolic pathway and debiased sparse partial correlation algorithm (DSPC) network analysis

The present study performed metabolic pathway analysis to identify any significant modulation caused by pathophysiological mechanisms associated with acclimatization to high altitude hypoxia in the Siachen group's metabolic pathway. For this purpose, the Pathway Analysis and Debiased Sparse Partial Correlation algorithm (DSPC) module of MetaboAnalyst 5.0^41^ (http://www.metaboanalyst.ca/) is used. The metabolic pathway is implemented with pathway impact values of more than 0.05 and a p-value less than 0.05. Each metabolite estimated in both baseline and Siachen group was matched with metabolites in a different pathway of the Kyoto Encyclopedia of Genes and Genomes (KEGG) database. Their statistical p-value is estimated, and the threshold for those having values less than 0.05. The topological analysis employing the out-degree centrality algorithm was used to estimate the pathway impact value while matching differential metabolites in metabolic pathways. Finally, ROC curve analysis is carried out to evaluate the pathway modulations. The area under the ROC curve (AUC) value was also estimated to evaluate the prediction performance in the disturbed metabolic pathway. In addition to the traditional metabolic pathway analysis, the present study explored the DSPC approach to understanding the metabolic alteration caused by the high-altitude hypoxic condition. This approach uses a de-sparsified graphical lasso modelling procedure and assumes that the number of the true association between the metabolites is much smaller.

## Results

### Demographic and clinical characteristics

The demographic and clinical data of the subjects were summarized in Fig. 1. As 30 participants were not included in the Siachen group due to extreme weather and logistic reasons, the Student t-test was carried out between the sea-level baseline and Siachen groups' demographic and clinical characteristics to find any group differences. The results revealed no significant difference in age, height, weight, and body mass index between the baseline and Siachen groups (P > 0.05).

### NMR spectra and metabolites concentration in urine samples

**Figure 2:**
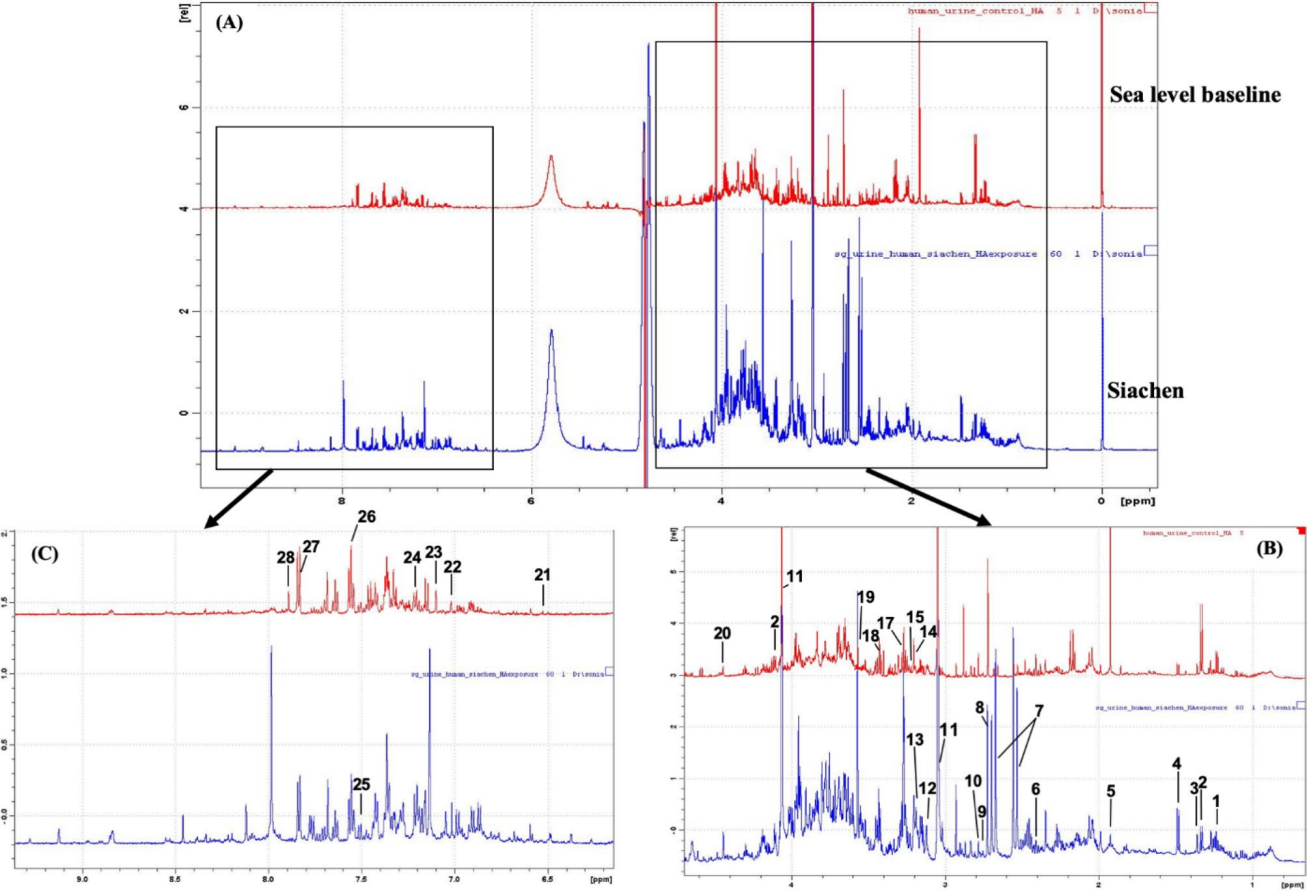
Representative 1H-NMR spectra (**A**) δ 0–δ 10.0 (**B**) δ 0.5–δ 4.5 (**C**) δ 6.5–δ 9.5, of urine samples from sea level baseline group and Siachen group. The labelled metabolites are: 1. Isoleucine, 2. Lactate, 3. Hydroxybutyrate, 4. Alanine, 5. Acetate, 6. Succinate, 7. Citrate, 8. Dimethylamine, 9. Sarcosine, 10. Dimethylglycine, 11. Creatinine, 12. Cysteine, 13. Cisaconitate, 14. Tyrosine, 15. Choline, 17. TMAO, 18. Acetoacetate, 19. Glycine, 20. NMN, 21. Fumarate, 22. Dihydroxymandalate, 23. Methylhistidine, 24. Tyrosine, 25. Indoxylsulphate, 26. Phenylalanine, 27. Hippurate, 28. Urocanate.

**Figure 3:**
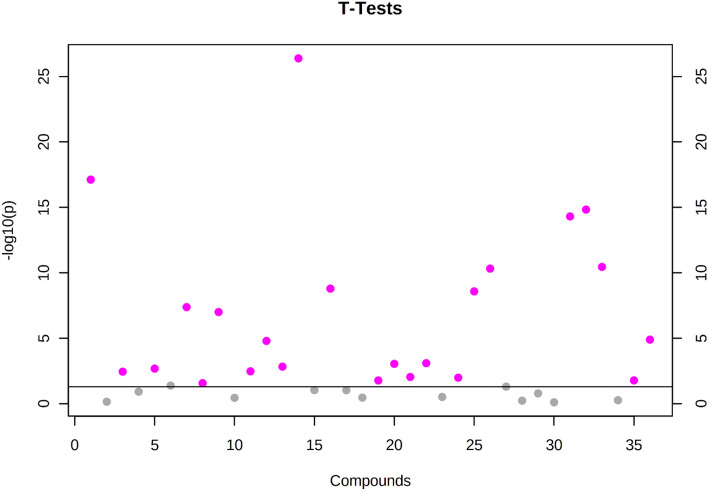
Important features selected by t-tests with threshold 0.1. The pink circles represent features above the threshold. Note the p values are transformed by − log10 so that the more significant features (With smaller p values) will be plotted higher on the graph.

A labelled 1H NMR spectra for sea-level baseline and Siachen group urine samples are shown in Fig. 2. Thirty-Six metabolites were identified, and their concentrations were estimated according to the literature. The univariate analysis between estimated metabolites from both groups revealed significant changes (p < 0.05 and FDR < 0.05) in twenty-three metabolites. Their statistical estimates are tabulated in Table 1 and shown in Fig. 3. Multivariate analysis and pathway analysis are carried out to bring more insight into these significantly modulated metabolites as the result of high-altitude hypoxia.

**Table 1:** List of 23 Key metabolites obtained from t-test responsible for discriminating sea level control group and Siachen group.

Metabolites name	Metabolite's concentration		Univariate analysis				Multivariate analysis			Pathway
	Area of metabolite's NMR peaks/area of NMR peak of 0.1 mM TSP (mean ± Std)									
	Baseline	Siachen	t-value	p value	− log10(p)	FDR	PLS-DA	OPLS-DA	Random forest	HMDB
	Sea-level	(Altitude: 3700 m)					VIP scores		MDA	
Cisaconitate	0.0544 ± 0.0150	0.3924 ± 0.0840	− 14.5560	0.0000	26.2080	0.0000	2.1597	2.0918	0.0490	HMDB0000072
NMN	0.0154 ± 0.0070	0.0456 ± 0.0088	− 12.5440	0.0000	21.8800	0.0000	1.8782	1.9151	0.1353	HMDB0000229
Tyrosine	0.3516 ± 0.1290	0.6681 ± 0.0909	− 9.4024	0.0000	14.8720	0.0000	1.7805	1.8007	0.0160	HMDB0000158
Choline	0.4881 ± 0.1544	0.7811 ± 0.0726	− 9.1794	0.0000	14.3720	0.0000	1.7560	1.8501	0.0266	HMDB0000097
Creatinine	5.0116 ± 1.6229	5.0499 ± 0.5427	7.6818	0.0000	11.0630	0.0000	1.5431	1.6007	0.0207	HMDB0000562
Isoleucine	0.4176 ± 0.1330	0.7659 ± 0.1244	− 7.5397	0.0000	10.7550	0.0000	1.5348	1.6129	0.0055	HMDB0000172
Alanine	0.2098 ± 0.0725	0.3218 ± 0.0319	− 6.6677	0.0000	8.9054	0.0000	1.4138	1.4630	0.0111	HMDB0000161
Cysteine	0.3519 ± 0.1147	0.3355 ± 0.0326	6.2969	0.0000	8.1432	0.0000	1.4294	1.3883	0.0075	HMDB0000574
Dihydroxymandalate	0.0556 ± 0.0189	0.0852 ± 0.0079	− 5.8819	0.0000	7.3118	0.0000	1.3182	1.3309	0.0033	HMDB0001866
Allantoin	0.0156 ± 0.0070	0.0298 ± 0.0035	− 4.9027	0.0000	5.4609	0.0000	1.2858	1.2422	0.0078	HMDB0000462
Acetoacetate	0.1341 ± 0.0485	0.1833 ± 0.0205	− 4.0759	0.0001	4.0491	0.0003	1.0650	1.0521	0.0107	HMDB0000060
Lactate	0.2661 ± 0.0863	0.3991 ± 0.0408	− 3.8325	0.0002	3.6647	0.0006	1.0761	1.0328	0.0180	HMDB0000190
Sarcosine	0.1906 ± 0.0617	0.2537 ± 0.0256	− 3.5301	0.0006	3.2089	0.0016	0.8341	0.8670	0.0028	HMDB0000271
TMAO	0.5334 ± 0.1812	0.5278 ± 0.0723	3.5292	0.0006	3.2076	0.0016	0.8007	0.7650	0.0028	HMDB0000925
Succinate	0.0428 ± 0.0131	0.0488 ± 0.0050	3.4421	0.0008	3.0809	0.0020	0.8418	0.8165		HMDB0000254
Indoxylsulphate	0.0338 ± 0.0152	0.0266 ± 0.0064	3.1382	0.0022	2.6563	0.0050				
Methylhistidine	0.0052 ± 0.0031	0.0040 ± 0.0013	2.8163	0.0058	2.2363	0.0123				
Dimethylamine	0.2874 ± 0.0879	0.3299 ± 0.0344	2.6066	0.0105	1.9799	0.0209				
Glycine	0.3636 ± 0.1567	0.5094 ± 0.0610	− 2.5009	0.0139	1.8560	0.0264				
Acetate	0.1145 ± 0.0455	0.1129 ± 0.0122	2.4159	0.0174	1.7589	0.0314				
Dimethylglycine	0.0921 ± 0.0309	0.1209 ± 0.0138	− 2.3599	0.0201	1.6962	0.0345				
Hydroxybutyrate	0.3158 ± 0.1073	0.3937 ± 0.0486	− 2.2830	0.0244	1.6118	0.0400				
Urocanate	0.0281 ± 0.0234	0.0166 ± 0.0180	2.2182	0.0287	1.5422	0.0449				

p values were derived from two-tailed Student’s t test. FDR values were calculated which indicates false discovery rates.

### Performance of multivariate analysis

**Figure 4:**
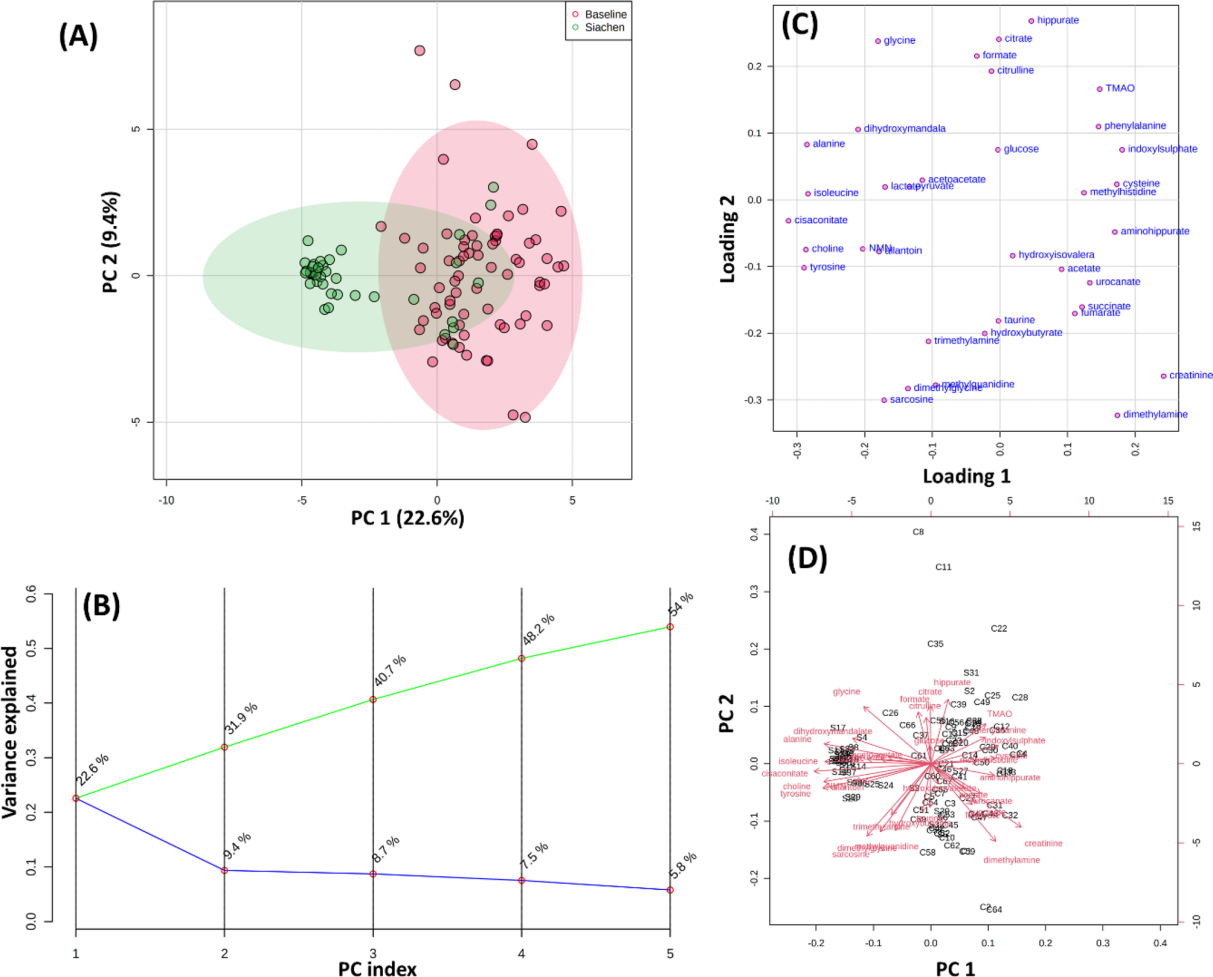
(**A**) PCA score plot between component 1 versus component 2 for 1H-NMR urine spectra from sea level Baseline group and Siachen group. (**B**) Scree plot was generated for the first five Principal components (PCs) to identify the contribution of each of the principal components to the total variation in the data set. The green line showed the cumulative variance explained by the first five components which accounted for 54% whereas blue line indicated the individual variance for each principal component. (**C**) Loading plots from PCA indicating the metabolites that lied far away from the origin and were responsible for separating the two groups. (**D**) PCA biplot between the selected PCs. Biplot for baseline and Siachen group identifying the relationship between samples and metabolites where samples were displayed as points and metabolites were displayed as vectors.

**Figure 5:**
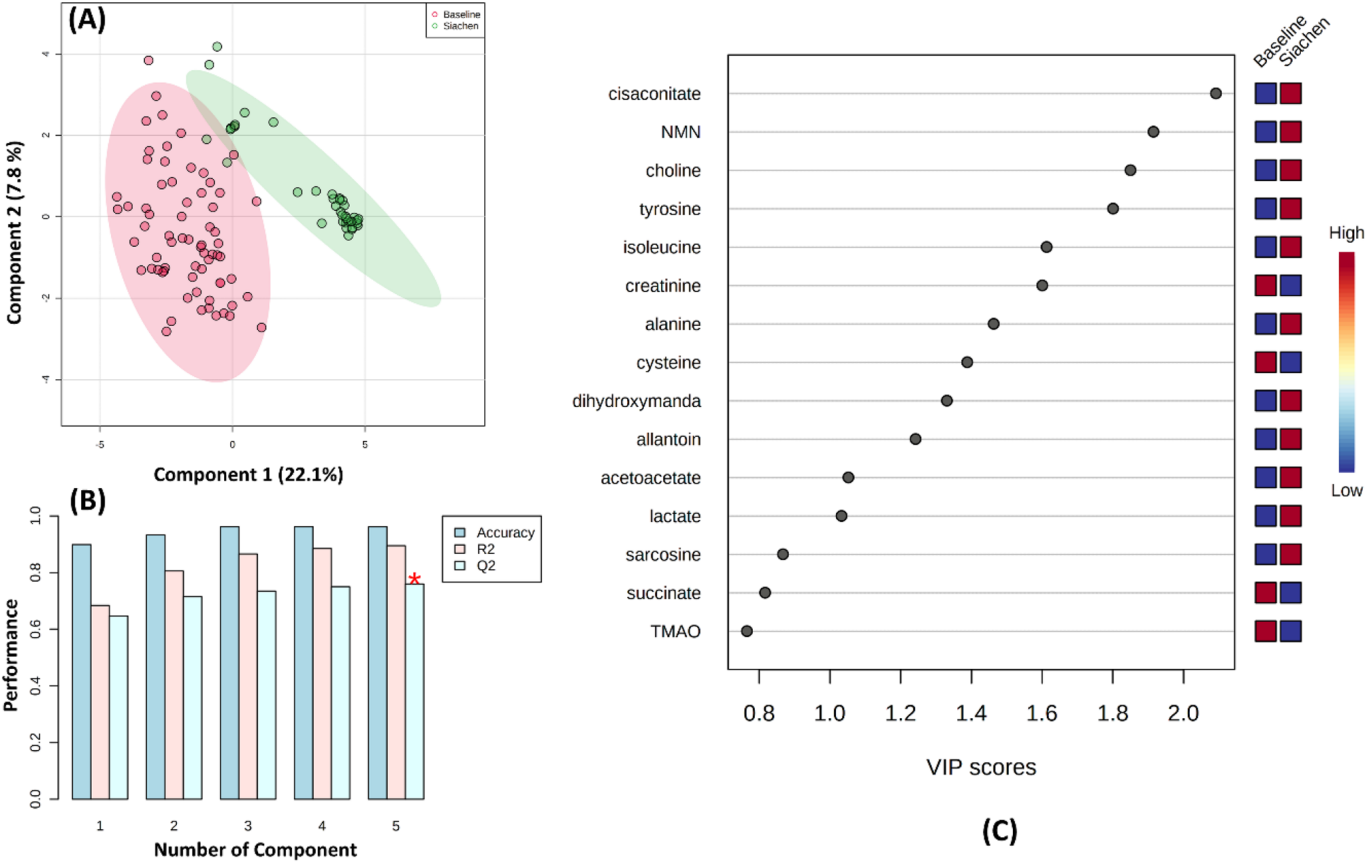
(**A**) PLS score plot between component 1 versus component 2 for 1H-NMR urine spectra from sea level baseline group (red open circle) and Siachen group (green open circle). (**B**) PLS-DA classification using different number of components. The red star indicates the best classifier. R2 and Q2, greater than 0.5 indicates a model with reasonable fit with good predictive power. The cross-validation analysis with first five components produced a strong model with high validated predictability (Q2 = 0.91), goodness of fit value (R2 = 0.78) and accuracy (accuracy = 0.96). (**C**) Based on the weighted sum of PLS-regression coefficients, the top 30 bins that contributed the maximum to separation of clusters in PLS-DA are shown. The coloured boxes on right show the relative concentrations of the corresponding metabolite.

**Figure 6:**
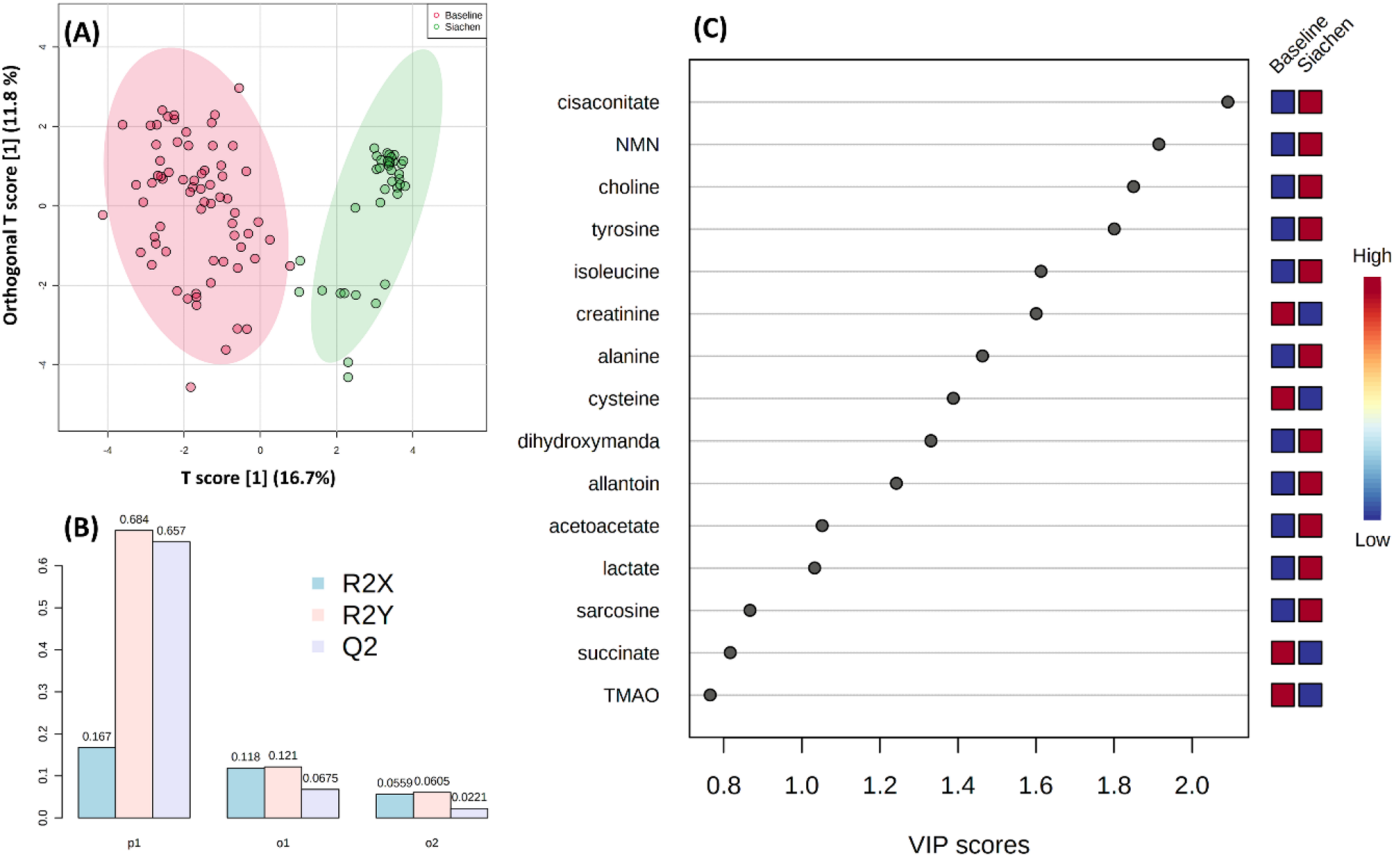
(**A**) OPLS-DA's score plot of all metabolite features between the sea-level baseline group and the Siachen group, (**B**) Cross-validated cumulative modelled variation R2X, R2Y, and Q2 coefficients of predictive loading (p1) and orthogonal (o1, o2) components (**C**) VIP scores of the significantly varying metabolites.

**Figure 7:**
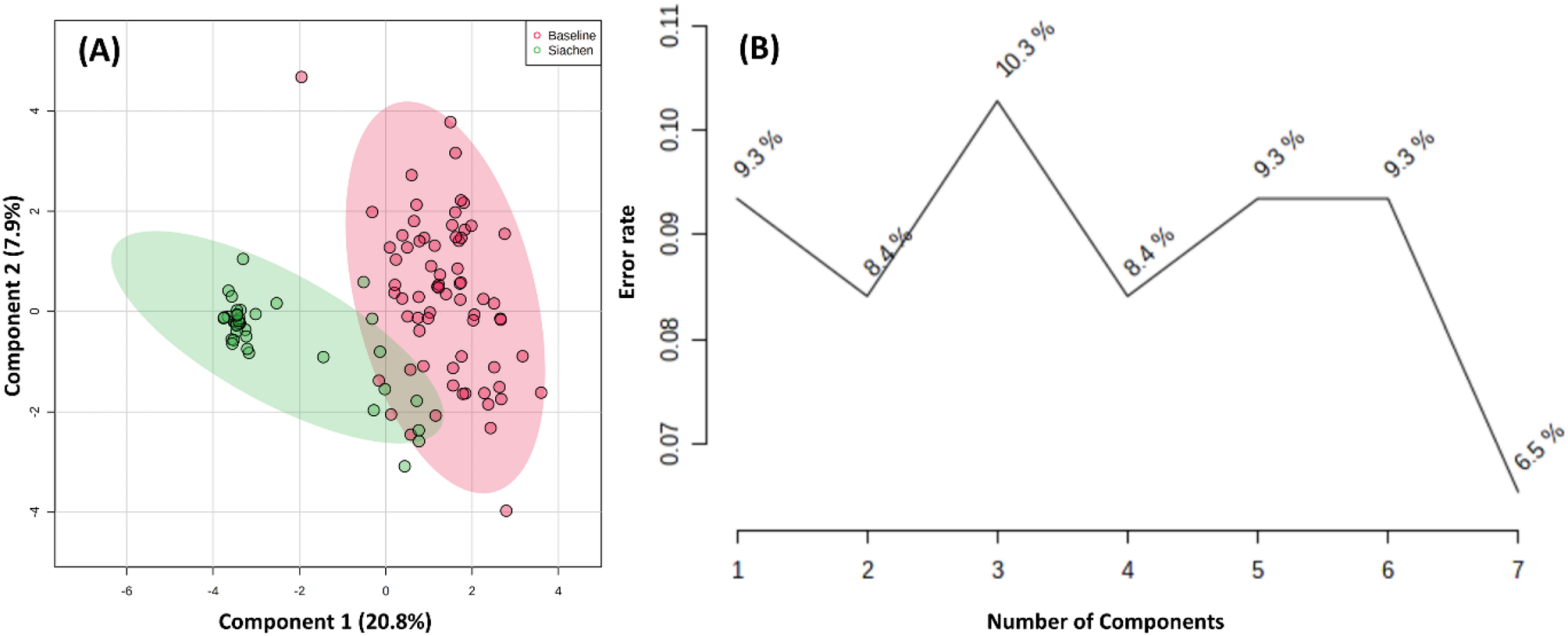
(**A**) sPLSDA score plots between selected PCs showing supervised clustering and separation between the sea-level control and Siachen groups, (**B**) Mean classification error rate for each sPLS-DA component.

Various multivariate statistical analysis was carried out to differentiate between the baseline and high-altitude hypoxia group, which further assessed the capability of the 1H NMR based metabolomics approach for early prediction in such scenarios. The principal component analysis (PCA) score plot showed a clear separation between the baseline and Siachen group (Fig. 4A). Loading plots from PCA (Fig. 4C) were generated to identify the metabolites that lied far away from the origin and were responsible for separating the two groups. The PCA Biplot for sea-level baseline and Siachen group (Fig. 4D) gave an overview between samples and metabolites where samples were displayed as points and metabolites were displayed as vectors. As shown in Fig. 4B, a Scree plot was generated for the first five principal components (PCs) to identify the contribution of each of the principal components to the total variation in the data set. The green line showed the cumulative variance explained by the first five components, which accounted for 54%, whereas the blue line indicated the individual variance for each principal component. PLS-DA revealed the most discriminate variables responsible for the clustering pattern (Fig. 5A). PLS-DA model quality parameters, R2 and Q2, more significant than 0.5, indicate a model with a good fit with good predictive power. The cross-validation analysis with the first five components produced a robust model with high validated predictability (Q2 = 0.91), the goodness of fit value (R2 = 0.78) and accuracy (accuracy = 0.96) (Fig. 5B). PLS-DA detected fifteen significantly different metabolites based on VIP score > 1 (Fig. 5C). These data were further used to perform OPLS-DA and sPLS-DA. Figure 6A shows the OPLS-DA's score plot of all metabolite features between the sea-level baseline group and the Siachen group, the VIP scores of the significantly varying metabolites (Fig. 6C), and the cross-validated cumulative modelled variation R2X, R2Y, and Q2 coefficients of predictive loading (p1) and orthogonal (o1, o2) components (Fig. 6B). The cumulative modelled variation describes the quality of the models in the *X* matrix, *R*2*X*, the cumulative modelled variation in the *Y* matrix, *R*2*Y*, and the cross-validated predictive ability or *Q*2 values. As is observed in Fig. 6B, the model is robust, with *Q*2 > 40% and *R*2Y > 50%. Figure 7 presents sPLSDA score plots between selected PCs showing supervised clustering and separation between the sea-level control and Siachen groups (Fig. 7A) and the Mean classification error rate for each sPLS-DA component (Fig. 7B). As seen from the clustering and error plot, the model separates sea level and Siachen urine metabolites information very well.

**Figure 8:**
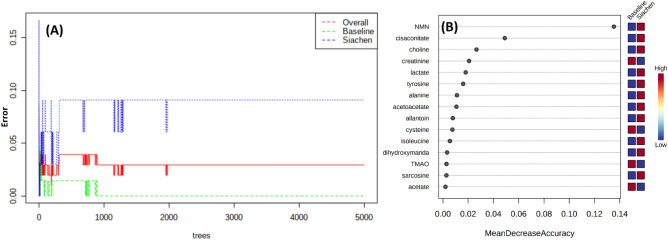
(**A**) Cumulative error rates by Random Forest classification. (**B**) Significant features identified by Random Forest. The features are ranked by the mean decrease in classification accuracy when they are permuted.

**Figure 9:**
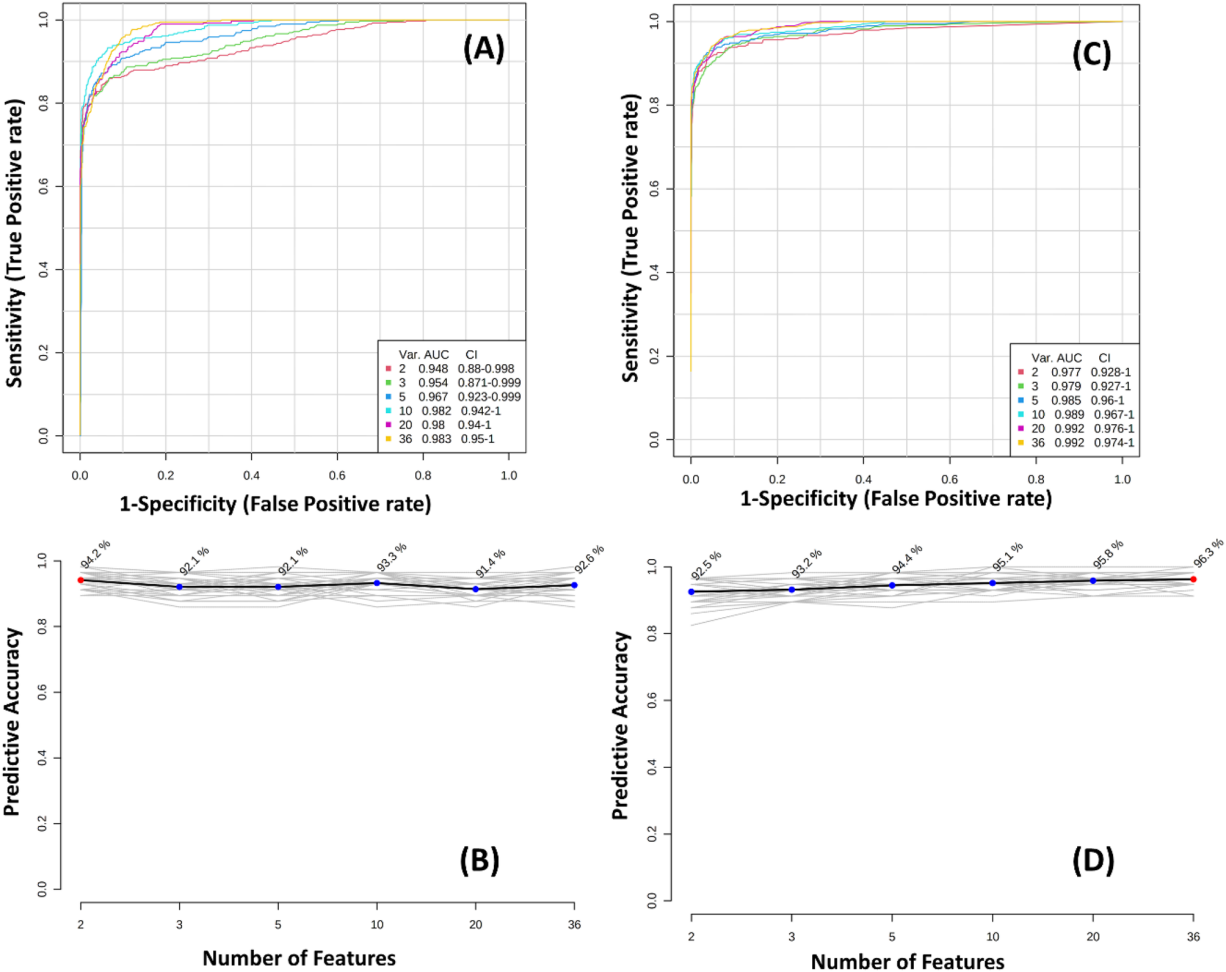
Receiver Operating characteristics (ROC) curve analysis and Predictive accuracies of different features of (**A**,**B**) PLS-DA and (**C**,**D**) Random Forest.

Finally, the random forest analysis was employed to understand the insights of metabolic modulation caused by the high-altitude hypoxia. Figure 8A plots the random forest algorithm's classification error plot and significant features (Fig. 8B) identified by Random Forest. The features are ranked by the mean decrease in classification accuracy when permuted. Five thousand trees were constructed, and proximities were estimated for the class as the tree was evolving. The significant metabolites observed in each multivariate analysis are shown in Table 1. Further, Receiver Operating characteristics (ROC) curve analysis was carried out to evaluate the performance of metabolites as biomarkers. The Fig. 9 depicts ROC curve analysis and Predictive accuracies of different PLS-DA (Fig. 9A,B) and Random Forest (Fig. 9C,D) approach features. As it is clear from the ROC analysis and AUC values, the random forest performs better than all other multivariate approaches.

### Metabolic pathway analysis for differentially expressed metabolites

**Figure 10:**
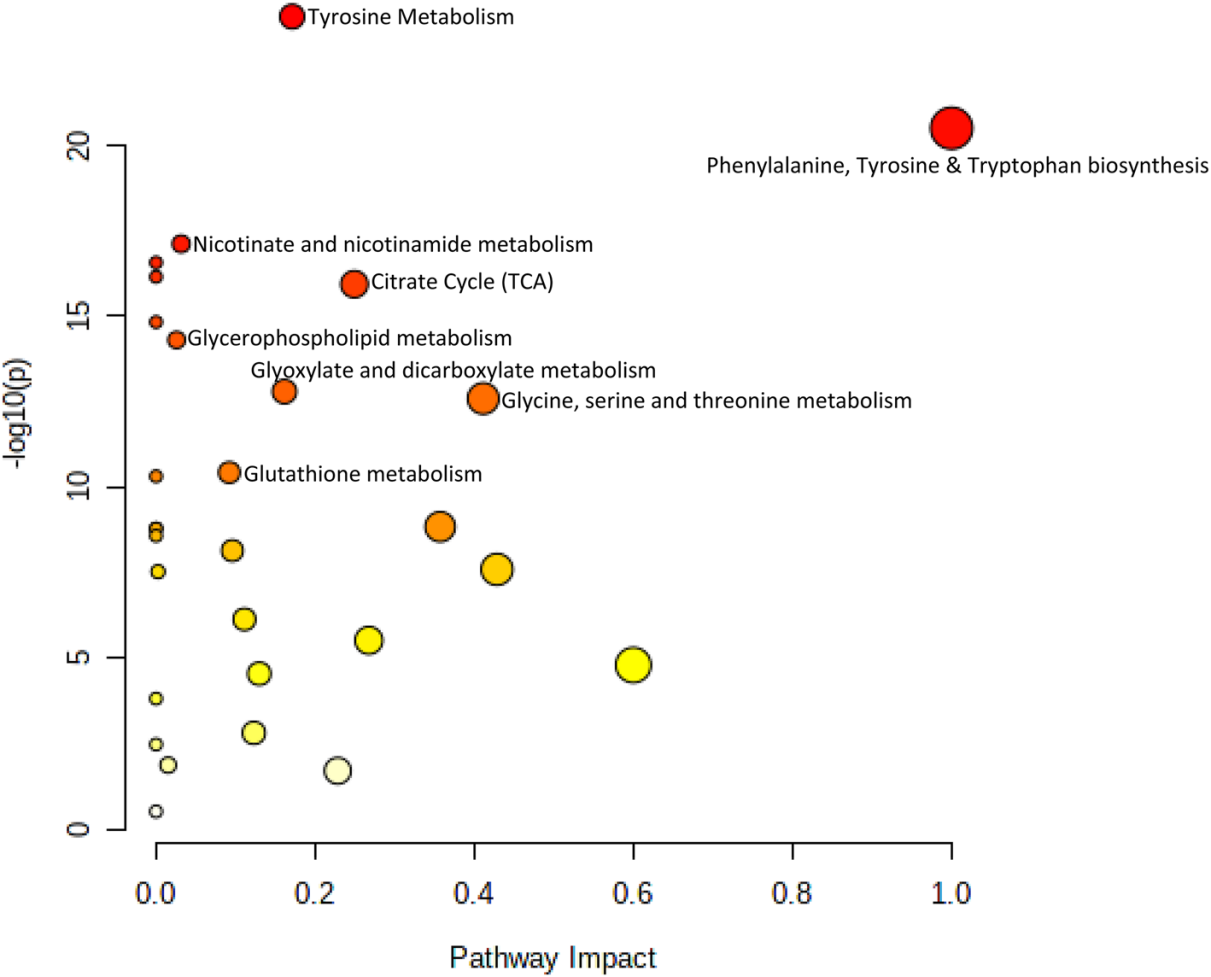
Summary of altered metabolic pathways in response to high altitude hypoxia environment.

This study employs metabolic pathways and Debiased Sparse Partial Correlation Network Modeling (DSPC) to get insights into altered metabolic pathways due to high altitude extreme environments. Out of 29 metabolic pathways analyzed, 16 pathways with pathway impact > 0.05 were considered in the pathway analysis. Phenylalanine, tyrosine and tryptophan biosynthesis were the most prominent pathways altered in extreme environments, with an impact value of 1. Succinate, cis-aconitate, citrate, pyruvate and fumarate were the metabolites of the TCA cycle out of 20 metabolites found to be altered between baseline and Siachen group (Fig. 10). KEGG databases from differential metabolites revealed 16 metabolic pathways between baseline sea level and the Siachen group, which were distinctly different. These metabolites were found to be primarily involved in Phenylalanine, tyrosine and tryptophan biosynthesis, TCA cycle, Tyrosine metabolism, Glyoxylate and dicarboxylate metabolism, Glutathione metabolism, Nicotinate and nicotinamide metabolism, Phenylalanine metabolism, Cysteine and methionine metabolism, Taurine and hypotaurine metabolism, Butanoate metabolism, Pyruvate metabolism, Synthesis and degradation of ketone bodies, Glycolysis/Gluconeogenesis, Histidine metabolism, Arginine biosynthesis (Table 2).

**Table 2:** KEGG Pathways of significantly different metabolites between sea level group and high-altitude Siachen group.

Id	Pathway name	Compound name	KEGG ID	HMDB
1	Phenylalanine, tyrosine and tryptophan biosynthesis	l-Phenylalanine	C00079	HMDB0000159
		l-Tyrosine	C00082	HMDB0000158
2	Glycine, serine and threonine metabolism	Choline	C00114	HMDB0000097
		N,N-Dimethylglycine	C01026	HMDB0000092
		Sarcosine	C00213	HMDB0000271
		Glycine	C00037	HMDB0000123
		Cysteine	C00097	HMDB0000574
		Pyruvate	C00022	HMDB0000243
3	Citrate cycle (TCA cycle)	Succinate	C00042	HMDB0000254
		Citrate	C00158	HMDB0000094
		cis-Aconitate	C00417	HMDB0000072
		Pyruvate	C00022	HMDB0000243
		Fumarate	C00122	HMDB0000134
4	Tyrosine metabolism	3,4-Dihydroxymandelate	C05580	HMDB0001866
		l-Tyrosine	C00082	HMDB0000158
		Fumarate	C00122	HMDB0000134
		Pyruvate	C00022	HMDB0000243
		Acetoacetate	C00164	HMDB0000060
5	Glyoxylate and dicarboxylate metabolism	cis-Aconitate	C00417	HMDB0000072
		Citrate	C00158	HMDB0000094
		Glycine	C00037	HMDB0000123
		Acetate	C00033	HMDB0000042
		Pyruvate	C00022	HMDB0000243
		Formate	C00058	HMDB0000142
6	Glutathione metabolism	Glycine	C00037	HMDB0000123
		l-Cysteine	C00097	HMDB0000574
7	Nicotinate and nicotinamide metabolism	Nicotinamide d-ribonucleotide	C00455	HMDB0000229
8	Phenylalanine metabolism	l-Phenylalanine	C00079	HMDB0000159
		Hippurate	C01586	HMDB0000714
		l-Tyrosine	C00082	HMDB0000158
9	Cysteine and methionine metabolism	Cysteine	C00097	HMDB0000574
		Pyruvate	C00022	HMDB0000243
10	Taurine and hypotaurine metabolism	l-Cysteine	C00097	HMDB0000574
		Taurine	C00245	HMDB0000251
11	Butanoate metabolism	Acetoacetate	C00164	HMDB0000060
		Succinate	C00042	HMDB0000254
12	Pyruvate metabolism	Pyruvate	C00022	HMDB0000243
		(S)-Lactate	C00186	HMDB0000190
		Acetate	C00033	HMDB0000042
		Fumarate	C00122	HMDB0000134
13	Synthesis and degradation of ketone bodies	Acetoacetate	C00164	HMDB0000060
14	Glycolysis/gluconeogenesis	Pyruvate	C00022	HMDB0000243
		(S)-Lactate	C00186	HMDB0000190
		beta-d-Glucose	C00221	HMDB0000122
		Acetate	C00033	HMDB0000042
15	Histidine metabolism	Urocanate	C00785	HMDB0000301
		N(pi)-Methyl-l-histidine	-	-
16	Arginine biosynthesis	l-Citrulline	C00327	HMDB0000904
		Fumarate	C00122	HMDB0000134

**Figure 11:**
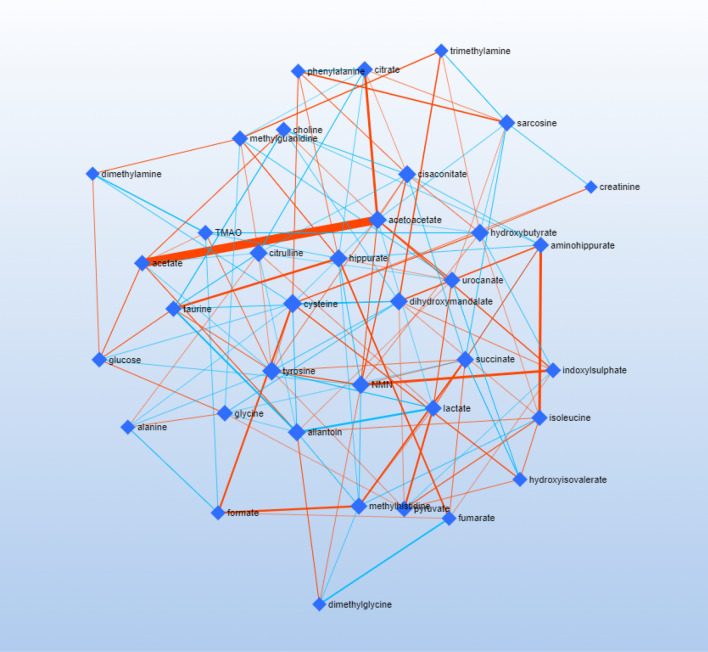
Debiased Sparse Partial Correlation Network Modelling (DSPC) indicating correlation between various metabolic features.

Debiased Sparse Partial Correlation Network Modeling (DSPC) and Visualization using the Network Explorer Module were carried out to visualize partial correlation networks using a data-driven network approach. DSPC reconstructs a network and calculates partial correlation coefficients and P-values for each pair of metabolic features. The results are visualized as a weighted network. The thicker the edges, the stronger the correlation between various metabolites and pathways (The red edges represent positive correlations, while the blue edges represent negative correlations). Figure 11 shows the results, which can be visualized as weighted networks, where nodes represent the metabolic features, and the edges depict the correlations among them^42^.

## Discussion

In the current study, urinary metabolic profiling was carried out single time sampled between baseline and Siachen participants (3700 m). The urine collection sampling was performed after 1 year of stay in the base and Siachen. Multiple sampling would give better holistic information at different time points. However, this study aims to perform an unsupervised, non-targeted study to identify fingerprint markers. The primary objective was to understand metabolic responses under long-term adaptations/maladaptation in high altitude stress. The phenomenon observed is at a point where chronic acclimatization to environmental asphyxia has already happened. These metabolic alterations do not come back to baseline levels, and as observed in the various native populations, there could be genetic modifications to adapt to these conditions. Since our cohort is posted for a short duration and returns to sea level after that, there is reported high altitude de-acclimatization syndrome, which needs to be monitored. As human physiology responds differently to high altitude exposure, it is worthwhile to understand the long-term effects that might lead to illnesses later due to load over various metabolic pathways.

It is a well-known fact that travelling to elevation is associated with a risk of developing AMS, HACE and HAPE^43,44^. Hypobaric hypoxia elicits a series of physiological responses that are highly variable in humans^45–47^. For military personnel, high altitude sicknesses such as AMS, HAPE and HACE can compromise the occupational performance and pose serious health risks. The current screening method for these illnesses relies on physical examination, oxygen saturation, evaluation of symptoms etc. No reliable technique for early diagnosis is currently being used. The study of metabolic markers will effectively understand the pathophysiological mechanism, early diagnosis, prognosis, and effective treatment. Out of various Omics techniques, NMR based metabolomics has the potential to identify fingerprint markers for early diagnosis. LC–MS and NMR are the two most common metabolomics techniques, but both have advantages and disadvantages. LC–MS has advantages over sensitivity and several detectable metabolites and is better for a targeted approach. In contrast, limitations of the technique are average reproducibility, complex sample preparation, higher cost, and time per sample. NMR spectroscopy is the preferred platform for long-term or large-scale clinical metabolomics studies due to its relative ease of sample preparation, the ability to quantify metabolite levels, the high level of experimental reproducibility, high throughput, and inherently non-destructive nature. The challenges with the NMR technique are lack of sensitivity with concentrations of metabolites identified > 1 μM and a lesser number of metabolites identified. Since our study requires an untargeted non-destructive approach, NMR is the method of choice. The study's finding suggests levels of a wide variety of amino acids being altered during high altitude adaptation.

### Phenylalanine, tyrosine and tryptophan biosynthesis/tyrosine metabolism

Changes in amino acids reflected metabolic remodelling to meet the energy requirements. Amino acid concentration alteration might reflect the utilization of glucogenic substrates. Altitude exposure results in stress on the body to maintain oxygen driven energetic pathways and redox homeostasis. Tyrosine metabolism was found to be affected during high altitude adaptation. Reduction in ATP production due to inhibition of citrate cycle induced by high altitude hypoxia could lead to utilising branched-chain amino acids such as tyrosine as energy compensation. Phenylalanine, Tyrosine and Tryptophan biosynthetic signalling pathways were affected due to high altitude adaptation. Levels of Tyrosine increased which might be due to replenishment of insufficient energy supply to adapt to hypoxic stress. Phenylalanine metabolism was affected as it needs to be converted to Tyrosine which further helps regulate oxidative stress, immune response, and inflammation to protect against damage^48–50^.

### TCA cycle

It is known that hypoxia affects cellular ATP production through the downregulation of several TCA cycle enzymes and compromising electron transport chain complexes. Reduction in ATP production due to inhibition of the TCA cycle induced by high altitude hypoxia could utilise branched-chain amino acids as an energy resource. Cis-aconitate was altered, which is an intermediate in the TCA cycle. In the TCA cycle, citrate undergoes stereospecific isomerization to isocitrate by the enzyme aconitase hydratase and intermediate cis-aconiate. Acotinase affects the conversion of citrate to iso-citrate through an intermediate cis-aconitate. Itaconate metabolite is derived from TCA cycle intermediate cis-aconiate, which is identified as an anti-inflammatory signal and shown to upregulate cellular antioxidant defences via induction of Nrf2^51–54^. During acute settings such as perinatal asphyxia, ischaemia–reperfusion, mechanical asphyxia, there is a complete cut down of oxygen and metabolism is driven towards the TCA cycle to satisfy ATP request^55–57^. However, in the case of high-altitude hypoxia, the partial pressure is reduced & during the long-term exposure, body adapts by slowing down the TCA cycle & ATP demand. In contrast, the energy requirement is compensated by BCAA & other breakdowns/replenishment mechanisms.

### Glycine, serine, and threonine metabolism

Glycine, Serine, and Threonine are essential amino acids for various amino acid metabolism pathways. Serine is made from phosphoglycerate and degraded to pyruvate, whereas glycine is made from serine with multiple degradation pathways. Both the TCA cycle and amino acid metabolism are linked. There is a reduction in ATP production due to inhibition of the TCA cycle induced by high altitude hypoxia, leading to the utilization of branched-chain amino acids as energy resource^2,18,22^.

A large part of cholines in biological systems occur in the form of immobile phospholipids and cell membranes. It forms the major precursor of neurotransmitters and cellular membranes^58,59^. The choline and macromolecule alterations may indicate compromised cell membrane metabolism or cellular damage. Choline levels were altered in response to high altitude adaptation, which gives insights into the altered metabolism of cholines and high choline demands in the cells and brain.

Creatinine is a waste product that the muscles produce at a steady rate as a normal daily activity. The bloodstream carries creatinine to the kidneys, which filter it out of the blood through urine. The kidneys play an essential role in human adaption to high altitude, during acclimatization and in mountain sickness illnesses by helping regulate body fluids, electrolytes and acid–base homeostasis. Creatinine levels were found to be altered at high altitudes due to reduced glomerular filtration rate, which was the result of changes in tissue oxygenation, renal blood flow and adaptive response of renal tissue. At chronic high-altitude exposure, vascular remodelling or enlargement was observed, which resulted in increased erythropoietin production, thereby increasing the red blood cell mass and haemoglobin to improve the oxygen-carrying capacity of the blood. The magnitude and duration of exposure to high altitude affect glomerular filtration rate, renal blood flow, renal plasma flow, and filtration fraction^60,61^.

Isoleucine, a branched-chain amino acid (BCAA) and primary mediator of alanine and glutamine biosynthesis increased during high altitude adaptation. BCAA catabolism begins in skeletal muscle with transamination of α-ketoglutarate yielding branched-chain ketoacids, which are further oxidised as succinyl co-A in the TCA cycle. BCAA plays a vital role in energy metabolism, and its increase indicates impaired energy metabolism resulting from oxidative stress and impaired mitochondrial respiration. During high altitude exposure, it is known that there is a loss of fat-free mass due to decreased physical activity, sleep cycle disruption, cold exposure, hypoxia, and changes in protein metabolism. As an adaptation to maintain muscle mass, there is an increase in BCAA isoleucine to compensate for the increased energy requirements at high altitude^18,62,63^.

Alteration in alanine metabolite was observed, a critical gluconeogenic α-amino acid delivering carbon from amino acid degradation in peripheral tissues and skeletal muscles^64,65^. Alanine is constituent of all proteins/peptides, and it can act as energy fuel via pyruvate oxidation released by transamination of alanine. Alanine gets converted to biomolecules such as pyruvate, 2-oxoglutrate and fumarate, which enter the TCA cycle to meet the ATP depletion and energy requirements, further promoting high altitude acclimatization.

### Glutathione metabolism and cysteine alterations

Glutathione is the essential component in antioxidant defence systems and measures the level of oxidative stress. It acts as a scavenger for free radicals. Regulation of Glutathione synthesis is highly dependent on intracellular availability of l-cysteine. During high altitude exposure, there is an increase in free radical production which might require more glutathione which in turn is generated by cysteine, thereby resulting in the decreased level of cysteine^66,67^.

Lactate concentration was found to be altered with acclimatization to high altitude. This might be explained by the lactate paradox phenomena linked to muscle oxygen delivery and energy demand at high altitudes. This is related to upregulated control contributions from cellular ATP demand and supply pathways^68,69^.

Trimethylamine N-oxide (TMAO) is a vital gut microbe-dependent metabolite generated from choline, betaine and l-carnitine, which is metabolized to trimethylamine (TMA) through gut microbiota metabolism. Alterations in TMAO indicate disturbed gut microbiota at high altitude^70^.

Nicotinamide adenine dinucleotide (NAD+) levels in the body are associated with downregulation of energy production in mitochondria, oxidative stress, DNA damage, cognitive impairment and inflammatory conditions. NMN is the precursor of NAD+, and under hypoxic conditions, NMN can slow down this process by elevating NAD+ levels in the body. Hence, an alteration in NMN levels was observed under long-term high-altitude hypoxia to prevent the damage.

### Challenges and limitations of the study

The present study aims to identify early predictive urinary metabolic biomarkers adaptations/maladaptation in human participants with long term exposure to high altitudes. One of the challenges while conducting this study was to manage the participants to stay in those extreme environments for a longer time, including extreme winter and other climatic, logistic conditions. Hence, this present study limited its scope in collecting the samples at limited time points with limited participants populations. However, further studies aiming to bring a more comprehensive, systemic analysis of high-altitude acclimatization with a much larger population and longitudinal specimen screening during the more extended stay will immensely benefit the research that aims to get the solutions to most the high-altitude illness.

## Conclusion

The present study is the first to profile the systemic metabolic alterations due to chronic high-altitude hypoxia in humans using NMR-based metabolism. Results unveil the metabolic pathways that play a crucial role in high altitude adaptations and need to be monitored. The study's outcome demonstrates NMR metabolomics as a powerful platform for understanding metabolic adaptations and maladaptation due to progressive altitude exposure. It is evident from the findings that cis-aconitate, Nicotinamide Mononucleotide, Tyrosine, Choline and Creatinine can be potential fingerprint biomarkers for early detection of high-altitude hypoxia-related illnesses. The outcome and results of the current study demonstrate the potential of metabolomics to provide detailed information on physiological adaptations, early diagnosis, prognosis, and effective treatment for high altitude maladies.

## Data Availability

The data that support the findings of this study are available on request from the corresponding author Dr Sonia Gandhi. The data are not publicly available due to DRDO being part of Ministry of Defence, Government of India, and sharing data through the FTP server is not allowed for security reasons.

## Abbreviations

1H NMR: Proton nuclear magnetic resonance spectroscopy
HH: Hypobaric hypoxia
HAPE: High-altitude pulmonary edema
HACE: High-altitude cerebral edema
AMS: Acute mountain sickness
TSP: Trimethylsilyl-2,2,3,3-tetra-deuteropropionic acid
D2O: Deuterium oxide
TXI: Triple resonance probe
FID: Free induction decay
HMDB: Human metabolome database
PR: Pattern recognition
PCA: Principle component analysis
PLS-DA: Partial least squares discriminant analysis
OPLS-DA: Orthogonal projections to latent structures discriminant analysis
VIP: Variable importance in projection
ROC: Receiver operating characteristic
sPLSDA: Sparse partial least square discriminant analysis
PC: Principle component
AUC: Area under the curve
TCA: Tricarboxylic acid
ATP: Adenosine triphosphate
BCAA: Branched-chain amino acid
TMAO: Trimethylamine N-oxide
HMDB: Human metabolome database
NOESY: Nuclear overhauser effect spectroscopy
